# Prevalence of human papillomavirus genotypes in Latvia among women participating in cervical cancer screening

**DOI:** 10.3389/fonc.2025.1584677

**Published:** 2025-06-30

**Authors:** Anna Stasulane, Justine Grundmane, Kristiana Sulte, Janis Stasulans, Solvita Cernavska, Laura Smite

**Affiliations:** ^1^ Faculty of Medicine, Riga Stradins University, Riga, Latvia; ^2^ E. Gulbis Laboratory, Riga, Latvia; ^3^ Faculty of Medicine and Life Sciences, University of Latvia, Riga, Latvia; ^4^ Riga Maternity Hospital, Riga, Latvia

**Keywords:** HPV, cervical cancer, genotypes, distribution, Latvia

## Abstract

**Background and objectives:**

The propensity of human papillomavirus (HPV) to cause cervical cancer is linked to specific genotypes that vary in carcinogenicity. This study aims to provide insight into the most prevalent HPV genotypes in Latvia among women participating in cervical cancer screening.

**Materials and methods:**

The prevalence data presented in this study were derived from routine screening. The data were collected during the first two years of the reorganized screening program, from 1 July 2022 to 1 July 2024, which for the first time included HPV genotyping as a primary screening method in women aged 30–70. Testing was performed in an accredited laboratory using a commercial kit capable of detecting 14 HPV genotypes.

**Results:**

The overall prevalence among 30–70-year-old women participating in cervical cancer screening in Latvia was 12.04%. HPV 16 was the most prevalent HPV genotype, followed by HPV 68, 31, 66, 52, 56, 51, 45, 33, 39, 18, 58, 59, and 35. Across all age categories, single infections were the most prevalent—81.82% of women tested positive for only one HPV genotype, with an average of 1.23 genotypes per positive sample. The prevalence of both single and co- infections tended to decrease with age, except for a slight increase in the oldest age group, women aged 60–70. There was a significant difference in the overall HPV infection prevalence across age groups.

**Conclusions:**

These results provide valuable epidemiological data that can inform cervical cancer screening, prognosis, HPV vaccine implementation targeting region-specific genotypes, and ongoing monitoring of genotype distribution.

## 1 Introduction

Persistent infection with oncogenic human papillomavirus (HPV) is a well-established risk factor for cervical cancer (1). Cervical cancer ranks as the fourth most common malignancy among women, with both high incidence and mortality rates. In 2022 alone, there were 660,000 new cases of cervical cancer globally, resulting in 350,000 deaths (2). Latvia ranks among the European Union countries with the highest incidence and mortality rates (3). The ability of HPV to cause cervical cancer is associated with specific genotypes (4). Identifying the most prevalent genotypes is crucial for designing effective screening strategies. This study aims to identify the most prevalent HPV genotypes among women in Latvia undergoing cervical cancer screening.

The history of cervical cancer screening in Latvia provides context for the current prevention efforts. Opportunistic cervical cancer screening was first introduced in occupied Latvia during the 1960s but was discontinued in 1991. After regaining independence, the first efforts to establish the cervical cancer screening program in the Republic of Latvia began in 2005, leading to its thorough reorganization in 2009 (5). Currently, the National Health Service sends invitation letters to either the declared or electronic address of women aged 25, 28, 31, 34, 37, 40, 43, 46, 49, 52, 55, 58, 61, 64, and 67, allowing them to participate in the screening program free of charge within three years. Although no formal reminder letters are sent to women who miss their screening appointments, healthcare providers—including gynecologists and family doctors—consistently recommend and encourage participation in cervical cancer screening during routine consultations and medical visits. Starting in July 2025, women over 30 will receive screening invitations every five, aligned with the negative HPV test schedule.

Examining the methodologies employed in screening can shed light on their effectiveness. Since 2009, cervical cancer screening in Latvia has involved a cytological smear, with targeted biopsies taken during colposcopy when necessary. The traditional method utilized conventional cytology, stained using the Romanowsky–Leishman method, differing from the standardized Papanicolaou stain used in most of Europe (6).

A significant shift occurred with the introduction of a new screening algorithm on 1 January 2019, which added a high-risk HPV test for certain cytological findings, such as ASC-US, LSIL, or AGUS. The test identified 14 high-risk HPV types but did not distinguish between them, instead determining only the presence or absence of HPV. Positive results prompted targeted biopsies during colposcopy, while negative results allowed patients to return to the routine screening program. This algorithm also marked a gradual transition from conventional to liquid-based cytology.

In pursuit of improved preventive care, the screening process has undergone notable changes in recent years. On 1 July 2021, the cervical cancer screening process underwent further changes, with the primary method shifting to liquid-based cytology. In cases where cytology results indicated A2 (ASC-US), A3 (LSIL), or A5 (AGUS), an HPV test was performed on the same sample. A positive HPV result referred the patient to a specialist for colposcopy, with or without biopsy, while cases of HSIL and ASC-H were referred for colposcopy with biopsy, without HPV testing.

Building on these advancements, since 1 July 2022, a new approach has been implemented in which primary HPV genotype testing replaces cervical cytology for women aged 30 to 70. For women aged 25 to 30, liquid-based cervical cytology remains the primary screening method. This change aims to enhance the accuracy of cervical cancer screening and diagnosis. The management algorithm is based on the specific HPV genotype detected and the results of cytological assessment results. Women aged 30 to 70 who test negative for high-risk HPV are considered at low risk and are scheduled to receive their next screening invitation letter after five years. In contrast, women who test positive for HPV types 16 and/or 18—due to their high oncogenic potential—are referred directly for colposcopy without further triage. For women who test positive for other high-risk HPV genotypes, cytological triage is performed. If cytology reveals atypical squamous cells of undetermined significance or worse (ASC-US+), the woman is referred to colposcopy. If the cytology result is negative, a repeat cytology is conducted after 12 months. Should the 12-month follow-up indicate ASC-US+, the patient is referred for colposcopy. If the result remains negative, a second cytological follow-up is carried out another 12 months later—that is, 24 months after the initial high-risk HPV test. Women whose cytology remains negative at both follow-up points return to routine screening and will receive their next invitation according to the national schedule (7).

Evaluating response rates offers insight into the effectiveness of the screening initiatives. These data are essential for understanding how representative the study’s results are of the entire female population in Latvia aged 30 to 70. The overall response rate during our study period between 1 July 2022 and 1 July 2024, was 53.32% (8). It is important to note that, since cervical cancer screening invitations in Latvia are valid for three years, women who were screened between 1 July 2022, and 1 July 2024 may have received their invitations at any point during that period—even earlier. Consequently, participation rates within this timeframe do not necessarily correspond to responses to invitations issued during the same period.

Finally, it is important to consider the role of vaccination in cervical cancer prevention. The first HPV vaccine became available for use in 2006. Currently, three HPV vaccines are authorized in Europe—Gardasil, Gardasil-9, and Cervarix, all of which are registered in Latvia. All vaccines are recombinant, containing purified L1 proteins from the HPV capsid. Gardasil contains L1 proteins from HPV types 6, 11, 16, and 18; Gardasil 9 contains proteins from HPV types 6, 11, 16, 18, 31, 33, 45, 52, and 58; while Cervarix includes two types of HPV proteins—types 16 and 18 (9). All available HPV vaccines are approved for use in both males and females starting at age 9—ideally, vaccination should occur before first sexual intercourse (10). After age 27, it is recommended that potential benefits be discussed on a case-by-case basis. However, vaccination after age 27 is especially recommended for immunocompromised individuals and men who have sex with men.

In Latvia, state-funded HPV vaccination began in 2010. Initially, only females aged 12 and above were vaccinated using a three-dose schedule with a bivalent vaccine targeting HPV types 16 and 18 (Cervarix) (11). The program later expanded to include the quadrivalent vaccine (Gardasil) and, since January 2020, the 9-valent vaccine (Gardasil 9), which covers additional HPV types (12). Notably, in 2022, vaccination was extended to boys aged 12–14; as of 2023, both girls and boys aged 12–18 are eligible for state-funded vaccination (13). The National Immunization Council currently recommends two doses of the HPV vaccine, at least 6 months apart, for healthy individuals. For immunocompromised individuals, a three-dose schedule is recommended (14). Similarly, World Health Organization recommends a one- or two-dose schedule for females aged 9 to 20 years and a two-dose schedule for females over 21 years (15).

Despite these efforts, HPV vaccination coverage in Latvia remains low compared to that in other European countries (16). In 2023, among 12-year-olds, first-dose coverage was 60.8% for girls and 54.6% for boys, while second-dose coverage was 46.1% for girls and 38% for boys. By age 15, 58% of girls had received the first dose, and 46% had completed the vaccination course. No data are available on HPV vaccination coverage by age 15 in males (17). Since the introduction of the WHO Cervical Cancer Elimination Strategy in 2018, Latvia has shown one of the highest percentage increases in vaccine coverage rates; however, it is still not projected to achieve the target of 90% vaccine coverage even by the year 2040 (18).

This study is the first of its kind to examine multiple HPV genotype prevalence in Latvia based on data from the cervical screening program, highlighting the country’s ongoing efforts to combat cervical cancer through both screening and vaccination initiatives. The focus of this study is to provide insight into the most prevalent HPV genotypes in Latvia among women aged 30 to 70 who are participating in the cervical cancer screening program. The data are drawn from the first two years of the reorganized screening program, which now, for the first time, includes HPV genotyping as a primary screening method for women aged 30–70.

## 2 Materials and methods

### 2.1 Study population

The study population was drawn from Latvia, a country in the Baltic region of Northern Europe with a population of over 1.8 million (19). It consisted of outpatients from gynecological clinics across the country who participated in the cervical cancer screening program.

### 2.2 Research ethics

This is an observational study that does not involve interventional experiments and poses no threat to the personal safety of the subjects. The study was conducted in accordance with the Declaration of Helsinki and was approved by the Riga Stradins University Research Ethics Committee (number: 2-PĒK-4/195/2024, dated 13 February 2024).

### 2.3 HPV testing

The research was based on data obtained from an accredited clinical laboratory, the E. Gulbis Laboratory (LVS EN ISO 15189:2013, ISO/IEC 17025, Riga, Latvia) (Latvian National Accreditation Bureau, 2022), which provides laboratory services across Latvia.

Cervicovaginal samples were collected with a liquid-based collection method (BD SurePath™, BD, USA) and delivered to the E. Gulbis’ laboratory via courier service. All samples were logged in the “5M” laboratory information system and then sent to the molecular diagnostics department. The cervicovaginal samples were then analyzed using a multiplex real-time PCR assay (Anyplex™ II HPV HR Detection, Seegene Inc., South Korea). The genotypes included in this assay—HPV 16, 18, 31, 33, 35, 39, 45, 51, 52, 56, 58, 59, 66, and 68—were analyzed individually.

### 2.4 Statistical analysis

Data collection was performed using the E. Gulbis Laboratory information system, which stores anonymized clinical data from laboratory visits, accessed via MySQL database queries. Statistical analysis and graphical representations were conducted using Microsoft Office 365 Excel and IBM SPSS Statistics 28 (IBM Corp., Armonk, NY, USA). Confidence intervals were calculated using the Wilson score method without continuity correction (20). Data that did not yield valid test results were excluded. To assess age-dependent trends in HPV prevalence, participants were grouped into four by age of genotyping into the following categories: 30–<40, 40–<50, 50–<60, and 60–70 years. Descriptive statistics were used to summarize the study groups. The chi-square test was used to analyze differences in overall HPV prevalence of HPV across age groups. A one-sample binomial test was used to analyze the difference between single-genotype HPV infections and coinfections. Results were considered statistically significant at a P-value <0.05.

## 3 Results

### 3.1 Overall HPV infection prevalence

In this study, 81,469 samples were collected at the E. Gulbis Laboratory as part of the routine systematic cervical cancer screening program conducted between 1 July 2022 and 1 July 2024. During this two-years period, 9,810 women tested positive for HPV, corresponding to an overall prevalence of 12.04% in this age group (95% CI 0.1182 to 0.1227) (**Table 1**).

**Table 1:** Age-specific and overall HPV infection prevalence in women.

Age group	Total test count	Positive infection count	Positive infection proportion (%) with 95% CI
30–<40	25,029	3,941	15.75% (0.1530–0.1620)
40–<50	24,362	2,683	11.01% (0.1063–0.1141)
50–<60	18,722	1,789	9.56% (0.0914–0.0999)
60–70	13,356	1,397	10.46% (0.0995–0.1099)
**30–70**	**81,469**	**9,810**	**12.04%** (0.1182–0.1227)

**Figure 1:**
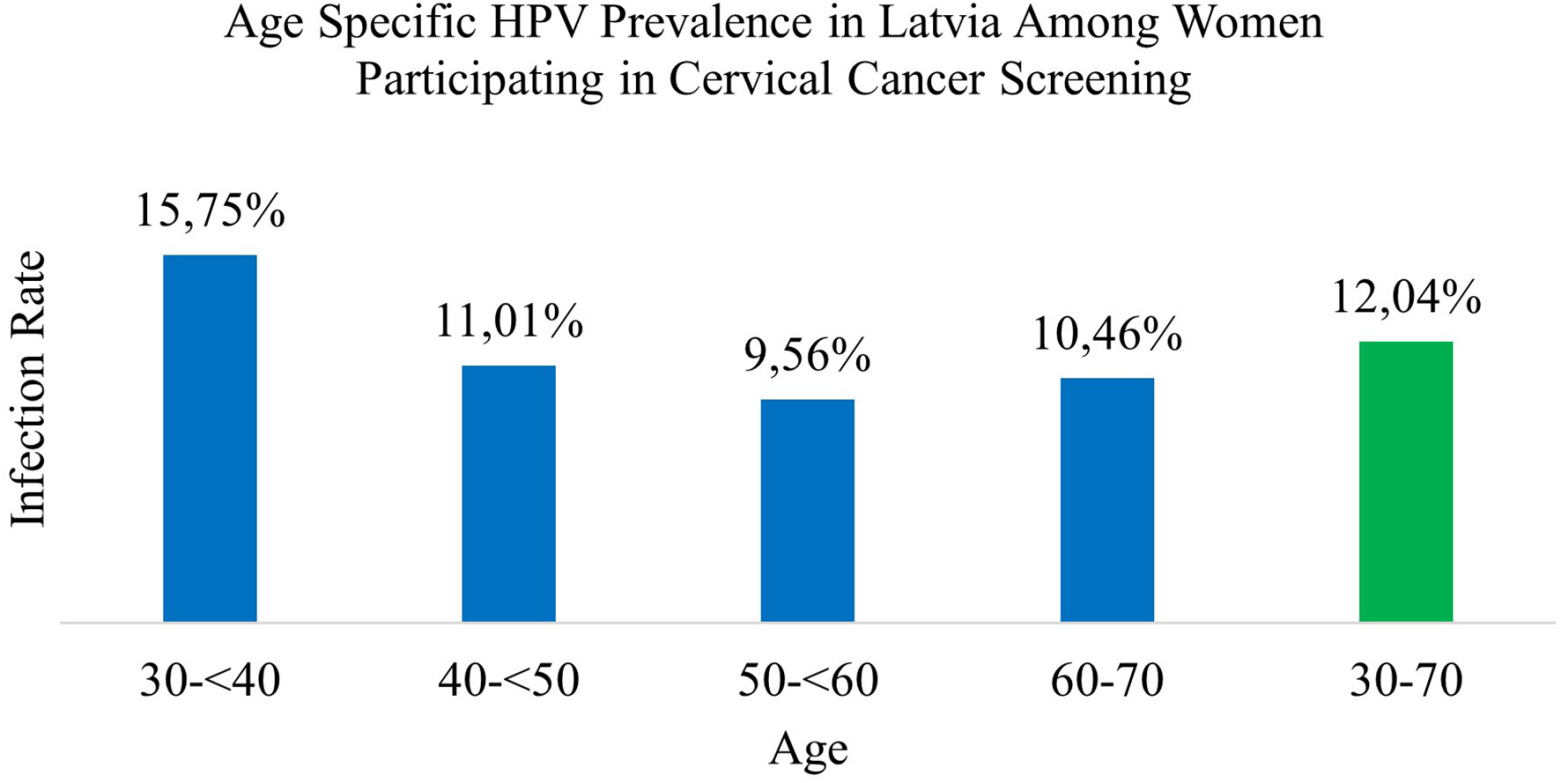
Age specific HPV prevalence in Latvia among women participating in cervical cancer screening.

Among these, 8,027 (81.82%) women tested positive for a single HPV genotype (95% CI 0.8105 to 0.8258) (**Table 2**). In comparison, 1,783 women (18.18%; 95% CI 0.1742 to 0.1895) tested positive for infections involving more than one HPV genotype. Among these, the majority had double infections (14.32%), followed by triple (3.04%), quadruple (0.09%), and quintuple infections (0.007%). Single HPV infections (95% CI 0.810 to 0.826) were significantly more prevalent than multiple genotype infections (p <0.001) (**Figure 1**).

**Table 2:** Age-specific distribution of single and multiple HPV infections among women aged 30–70 years.

Age group	Single infection count	Single infection proportion (%) with 95% CI	Multiple infection count	Multiple infection proportion (%) with 95% CI
30–<40	3,074	12.28 (0.1188–0.1269)	867	3.46 (0.0324–0.0370)
40–<50	2,276	9.34 (0.0898–0.0971)	407	1.67 (0.0152–0.0184)
50–<60	1,511	8.07 (0.0769–0.0847)	278	1.48 (0.0132–0.0167)
60–70	1,166	8.73 (0.0826–0.0922)	231	1.73 (0.0152–0.0196)
**30–70**	**8,027**	**9.85** (0.0964–0.1006)	**1,783**	**2.19** (0.0209–0.0229)

### 3.2 Genotype specific prevalence of HPV infection

In this comprehensive study, 14 HPV genotypes included in the screening program were analyzed. Among 9,810 HPV-positive cervical samples from women aged 30 to 70 years, a total of 12,051 genotypes were detected, corresponding to an average of 1.23 genotypes per positive sample (**Table 1**).

**Figure 2:**
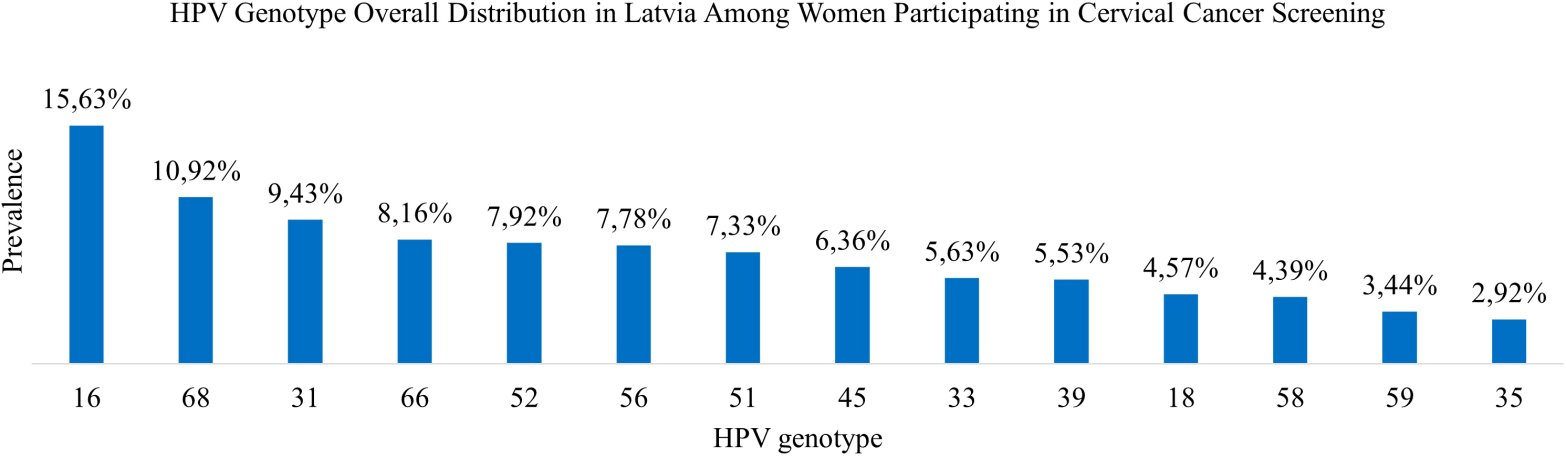
HPV genotype overall distribution in Latvia among women participating in cervical cancer screening.

HPV 16 was the most prevalent genotype, representing 15.63% of all detected HPV genotypes (95% CI: 0.1499–0.1628) and 19.19% of HPV-positive samples (95% CI: 0.1836–0.2003). HPV 68 was the second most common genotype comprising 10.92% of detected genotypes (95% CI: 0.1038–0.1147) and 13.41% of HPV-positive cases (95% CI: 0.1275–0.1410). HPV 31 ranked third, accounting for 9.43% of genotypes (95% CI: 0.0893–0.0997) and 11.59% of HPV-positive samples (95% CI: 0.1097–0.1224). HPV 66 followed, comprising 8.16% of genotypes (95% CI: 0.0768–0.0866) and 10.02% of HPV-positive cases (95% CI: 0.0944–0.1063). The remaining genotypes were less prevalent, each accounting for between 7.92% and 2.92% of the total genotype distribution (**Table 3**; **Figure 2**).

**Table 3:** Overall and age-specific HPV genotype distribution of detected genotypes among women.

Genotype	30–<40 count	30–<40 Proportion (%)	40–<50 count	40–<50 Proportion (%)	50–<60 count	50–<60 Proportion (%)	60–70 count	60–70 Proportion (%)	30–70 count	30–70 Proportion (%)
HPV 16	868	17.21	469	14.72	279	13.08	267	15.83	1,883	15.63
HPV 68	448	8.88	399	12.52	279	13.08	190	11.26	1,316	10.92
HPV 31	515	10.21	272	8.54	181	8.49	169	10.02	1,137	9.43
HPV 66	338	6.70	264	8.29	204	9.56	177	10.49	983	8.16
HPV 52	445	8.82	244	7.66	152	7.13	114	6.76	955	7.92
HPV 56	338	6.70	272	8.54	187	8.77	141	8.36	938	7.78
HPV 51	396	7.85	235	7.38	138	6.47	114	6.76	883	7.33
HPV 45	293	5.81	232	7.28	145	6.80	96	5.69	766	6.36
HPV 33	299	5.93	169	5.30	115	5.39	95	5.63	678	5.63
HPV 39	303	6.01	163	5.12	123	5.77	77	4.56	666	5.53
HPV 18	263	5.21	141	4.43	95	4.45	52	3.08	551	4.57
HPV 58	203	4.02	137	4.30	109	5.11	80	4.74	529	4.39
HPV 59	174	3.45	106	3.33	73	3.42	61	3.62	414	3.44
HPV 35	162	3.21	83	2.61	53	2.48	54	3.20	352	2.92

### 3.3 Age-specific infection and co-infection patterns

Among women aged 30–<40 years, 15.75% tested positive for HPV. The majority had single-genotype infections (12.28%), while co-infections were identified in 3.46% of cases. Double infections were the most frequent (2.69%), followed by triple (0.62%), quadruple (0.14%), and quintuple infections (0.016%).

In the 40 to <50 age group, the HPV positivity rate was 11.01%. Single-genotype infections accounted for 9.34%, and co-infections for 1.67%, comprising 1.33% for double infections, 0.28% with triplet, and 0.07% with quadruple infections. Quintuple infections were not observed in this age group.

Among women aged 50 to <60 years, 9.56% tested positive for HPV, including 8.07% with single-genotype infections and 1.48% with co-infections. Double infections comprised 1.20%, followed by triple (0.22%), quadruple (0.06%), and quintuple (0.005%) infections.

In the 60 to 70-year age group, 10.46% tested positive for HPV. Of these, 8.73% had single-genotype infections and 1.73% had co-infections, including 1.39% with double, 0.25% with triple, 0.08% with quadruple, and 0.007% with quintuple infections.

Overall, single-genotype infections were more prevalent than co-infections across all age groups. Infection rates generally declined with age, although a slight increase was observed in the 60 to 70-year group. The differences in overall HPV prevalence by age were statistically significant (χ² = 489.370, *p <*0.001) (**Tables 2**, **4**; **Figure 1**).

**Table 4:** Prevalence of multiple HPV infections by age group.

Age group	Double infections	Double proportion (%) with 95% CI	Triple infections	Triple proportion (%) with 95% CI	Quadruple infections	Quadruple proportion (%) with 95% CI	Quintuple infections	Quintuple proportion (%) with 95% CI
30–<40	673	2.69 (0250–0.0290)	154	0.62 (0.0053–0.0072)	36	0.14 (0.0010–0.0020)	4	0.016 (0.0004–0.0010)
40–<50	323	1.33 (0.0119–0.0148)	68	0.28 (0.0022–0.0035)	16	0.07 (0.0011–0.004)	0	0.000
50–<60	224	1.20 (0.0105–0.0136)	42	0.22 (0.0017–0.0030)	11	0.06 (0.0003–0.0011)	1	0.005 (0.0000–0.0003)
60–70	185	1.39 (0.0120–0.0160)	34	0.25 (0.0018–0.0036)	11	0.08 (0.0005–0.0015)	1	0.007 (0.0000–0.0004)
**30–70**	**1,405**	**1.72** (0.1364–0.1503)	**298**	**0.37** (0.0272–0.0340)	**74**	**0.09** (0.0007–0.0011)	**6**	**0.007** (0.0000–0.0001)

### 3.4 Age-specific genotype distribution

Among women aged 30 to <40 years, HPV 16 was the most frequently detected genotype (17.21%), followed by HPV 31 (10.21%) and HPV 68 (8.88%). Other commonly detected types included HPV 52 (8.82%), HPV 51 (7.85%), HPV 66 and HPV 56 (each 6.70%), HPV 39 (6.01%), HPV 33 (5.93%), and HPV 45 (5.81%). Less prevalently genotypes were HPV 18 (5.21%), HPV 58 (4.02%), HPV 59 (3.45%), and HPV 35 (3.21%) (**Table 3**).

In the 40 to <50-year age group, HPV 16 remained the most prevalent genotype (14.72%), followed by HPV 68 (12.52%). HPV 31 and HPV 56 were each detected in 8.54% of samples, while HPV 66 (8.29%), HPV 52 (7.66%), HPV 51 (7.38%), and HPV 45 (7.28%) were frequently observed. Additional genotypes included HPV 33 (5.30%), HPV 39 (5.12%), HPV 18 (4.43%), HPV 58 (4.30%), HPV 59 (3.33%), and HPV 35 (2.61%) (**Table 3**).

Among women aged 50– to <60 years, HPV 16 and HPV 68 were equally prevalent, each accounting for 13.08% of detections. These were followed by HPV 66 (9.56%), HPV 56 (8.77%), HPV 31 (8.49%), and HPV 52 (7.13%). Other frequently detected genotypes included HPV 45 (6.80%), HPV 51 (6.47%), HPV 39 (5.77%), and HPV 33 (5.39%). Less common types were HPV 58 (5.11%), HPV 18 (4.45%), HPV 59 (3.42%), and HPV 35 (2.48%) were less common (**Table 3**).

In the 60 to 70-year age group, HPV 16 remained the most prevalent genotype (15.83%), followed by HPV 68 (11.26%) and HPV 66 (10.49%). HPV 31 (10.02%) and HPV 56 (8.36%) were also frequently detected. HPV 52 and HPV 51 were each identified in 6.76% of samples, followed by HPV 45 (5.69%), HPV 33 (5.63%), HPV 58 (4.74%), and HPV 39 (4.56%) followed. The least prevalent genotypes in this group were HPV 59 (3.62%), HPV 35 (3.20%), and HPV 18 (3.08%) (**Table 3**).

## 4 Discussion

In this large population-based study of women aged 30 to 70 years undergoing routine cervical cancer screening in Latvia, the overall prevalence of HPV was 12.04%. Single-genotype infections were more prevalent than multiple-genotype infections across all age groups. Among high-risk HPV types, HPV 16 was the most common, followed by HPV 68, HPV 31, HPV 66, HPV 52, and HPV 56. Notably, HPV 68 and HPV 66 showed higher prevalence rates than those typically reported in other European studies, with HPV 68 ranking second and HPV 66 taking the fourth position among 14 tested genotypes, indicating potential regional differences in genotype circulation (21). The World Health Organization has suggested that the probably (Group 2A) and possibly (Group 2B) carcinogenic HPV types, including HPV 68 and HPV 66, should be excluded from screening tests in the future (22). Since the study sample included participants from a national screening program, these findings are likely representative of the broader female population in Latvia aged 30 to 70.

Previous studies on HPV prevalence in Latvia have reported varying genotype distributions compared to the present findings. These differences are primarily attributable to variations in study populations, research designs, and testing methods, rather than changes in HPV type circulation. All of these factors should be considered when comparing study results, as they may lead to variations across findings. These factors can influence detection rates and genotype prevalence, highlighting the need to report technical aspects in each study to ensure reliable comparisons.

HPV genotype prevalence in Latvia was first researched in a 2007 study (23) in which 442 women were tested for high-risk HPV genotypes: HPV 16, HPV 31, HPV 35, HPV 39, the combined group HPV 18 and HPV 45, and the combined group HPV 33, HPV 52, and HPV 58. At that time, the overall prevalence of these high-risk HPV genotypes in Latvia was 26.2%, with HPV 16 being the most common, followed by HPV 33 and HPV 39. The prevalence reported in the 2007 study (26.1%) contrasts sharply with that found in our study (12.04%). This difference should be interpreted with caution, given the variations in study design. Our study included only women aged 30 to 70 who participated in the cervical cancer screening program. In contrast, the 2007 study included women aged 15 to 85 who participated in voluntary cervical cancer screening, were gynecological outpatients, or were seen at sexually transmitted disease clinics—factors that may have contributed to higher HPV positivity rates.

A recent 2023 study (24) in Latvia involved a general population sample of 744 women who used a self-sampling method to collect cervical samples. The study found HPV 16 present in 3.5% of all samples, which is comparable to the 2.31% prevalence in our study (1,883/81,469). In the 2023 self-sampling study, HPV 18 was detected in 1.2% of samples, while in our study, it was present in 0.68% (551/81,469) of samples. Other high-risk types (HPV 31, HPV 33, HPV 35, HPV 39, HPV 45, HPV 51, HPV 52, HPV 56, HPV 58, HPV 59, HPV 66, HPV 6) were tested in the 2023 study using the genotype pooling method, which limits the ability to identify individual HPV genotypes, particularly in co-infections. These genotypes accounted for a 7.5% presence in all samples, and the overall prevalence in the 2023 study was 12.2%, closely aligning with the 12.04% prevalence found in our study involving 81,469 women.

A meta-analysis of women with normal cytology findings reported HPV prevalence rates of 21.4% in Eastern Europe, 10.8% in Northern Europe, 8.8% in Southern Europe, and 9.0% in Western Europe. Our results for women in Latvia participating in cervical cancer screening showed a lower prevalence of 12.04% HPV compared to the 14.2% European average. Globally, the five most prevalent HPV types among HPV-positive women are HPV 16, HPV 18, HPV 31, HPV 58, and HPV 52. However, the prevalence of these genotypes varies by region. In Europe, HPV 31 is particularly frequent (25), and our study also found a high prevalence of this genotype.

A study from Portugal (26) and a study from Italy (27) used similar methodologies and the same assay as our study (Anyplex™ II HPV HR Detection, Seegene Inc., South Korea). HPV16 was among the most prevalent types in all three countries, with Latvia (15.63%) showing a slightly lower prevalence than Portugal (17.5%) but a higher prevalence than Italy (13.8%). This suggests consistent circulation of this high-risk type across Europe. Latvia showed a generally narrower range of high-prevalence HPV types, with fewer genotypes exceeding 10% compared to Portugal, which had multiple types above that threshold. HPV68 holds a similar rank in Portugal (4th place), Italy (3rd place), and Latvia (4th place), indicating relatively consistent prevalence across these regions. HPV31 ranks among the top three most prevalent genotypes, with a prevalence of 15.0% in Portugal (3rd place), 12.9% in Italy (2nd place), and 9.43% in Latvia (3rd place), reinforcing its role as a common high-risk genotype across European populations. In contrast, HPV39 demonstrates significant regional variation, ranking 2nd in Portugal with a prevalence of 16.7%, but only 9th in Italy (5.3%) and 10th in Latvia (5.53%), suggesting a region-specific elevation of HPV39 in Portugal.

Many demographic and behavioral factors are known to affect the risk of HPV infection and persistence, which is an important consideration when interpreting the results from our study. A variety of risk factors for HPV infection and cervical carcinogenesis have been established in prior studies, including early sexual debut, higher lifetime number of sexual partners, use of oral contraceptives, smoking, and high parity (28). While our study did not collect behavioral data, previous research suggests that these factors may influence HPV prevalence patterns. Socioeconomic and geographical inequalities in Latvia may also affect exposure to these risk factors, potentially influencing HPV acquisition and clearance rates. Future studies involving individual-level risk factor data would help further inform the epidemiology of HPV infections in this population.

Since January 2020, girls aged 12–18 in Latvia have been vaccinated has been carried out with the 9-valent vaccine against HPV 6, HPV 11, HPV 16, HPV 18, HPV 31, HPV 33, HPV 45, HPV 52, and HPV 58. Our study indicates the current vaccine does not provide against HPV 68—the second most frequent genotype in Latvia—and the fourth most common overall, as well as other less common genotypes such as HPV 56, HPV 51, HPV 39, HPV 39, HPV 59, HPV 35. Our study cannot directly assess the effect of HPV vaccination on genotype distribution due to the lack of individual-level vaccination data.

Future studies should focus on longitudinal research that track the persistence of specific HPV genotypes and the long-term effects of vaccination. These studies will be crucial for monitoring genotype prevalence, evaluating the longevity of vaccine-induced immunity, and identifying potential type replacement. Ongoing surveillance will also help assess vaccine effectiveness across diverse populations and inform public health strategies aimed at optimizing HPV prevention efforts.

## 5 Limitations

As the HPV assay used in this study complied with World Health Organization recommendations for screening-prioritized types, only 14 HPV genotypes (HPV 16, HPV 18, HPV 31, HPV 33, HPV 35, HPV 39, HPV 45, HPV 51, HPV 52, HPV 56, HPV 58, HPV 59, HPV 66, and HPV 68) were analyzed as part of the cervical screening program, limiting the understanding of other potentially prevalent HPV infections. Future studies could explore other potentially relevant genotypes not included in the current screening test kit to better capture the full spectrum of HPV infections.

The two-year observation period in this study may be insufficient to capture longer-term trends in HPV genotype prevalence. A longer follow-up period would be necessary to better assess the potential impact of the HPV vaccination rollout, genotype distribution dynamics, and the persistence of different HPV types.

As this study focused exclusively on women participating in the national cervical cancer screening program, the findings may not fully reflect HPV genotype prevalence among women who did not participate. Efforts to reduce non-participation could enhance the representativeness of future studies.

## 6 Conclusions

This large, nationwide, screening-based study demonstrated an HPV prevalence of 12.04% among women participating in Latvia’s national cervical cancer screening program. The most prevalent genotypes were HPV 16, HPV 68, HPV 31, HPV 66, and HPV 52—of which HPV 68 and HPV 66 are currently not covered by the vaccine. Across all age categories, single infection was the most prevalent form— 81.82% of women tested positive for one HPV genotype, with an average of 1.23 genotypes per positive sample. The rates of both single HPV infections and co-infections tended to decrease with age, with a slight increase in the oldest age group (60–70 years). There was a significant difference in overall HPV prevalence across the age groups. These results provide substantial epidemiological evidence to inform cervical cancer screening strategies, guide HPV vaccination policies in this region, and support ongoing monitoring of HPV genotype distribution in Latvia.

## Data Availability

The raw data supporting the conclusions of this article will be made available by the authors, without undue reservation.
